# Comparing oxytocin and cortisol regulation in a double-blind, placebo-controlled, hydrocortisone challenge pilot study in children with autism and typical development

**DOI:** 10.1186/s11689-016-9165-6

**Published:** 2016-08-18

**Authors:** Blythe A. Corbett, Karen L. Bales, Deanna Swain, Kevin Sanders, Tamara A. R. Weinstein, Louis J. Muglia

**Affiliations:** 1 Department of Psychiatry and Behavioral Sciences, Vanderbilt University, PMB 40, 230 Appleton Place, Nashville, TN 37203 USA; 2 Vanderbilt Kennedy Center for Research on Human Development, Nashville, TN USA; 3 Department of Psychology, Vanderbilt University, Nasvhille, TN USA; 4 University of California, Davis, CA USA; 5 Virginia Polytechnic Institute and State University, Blacksburg, VA USA; 6 Department of Pediatrics, Perinatal Institute, Cincinnati Children’s Hospital Medical Center, University of Cincinnati College of Medicine, Cincinnati, OH USA

**Keywords:** Oxytocin, Cortisol, Hydrocortisone, Autism, Hormone, Stress, Arginine vasopressin, Stress, Autism spectrum disorder, LHPA axis

## Abstract

**Background:**

Children with autism spectrum disorder (ASD) show marked impairment in social functioning and poor adaptation to new and changing contexts, which may be influenced by underlying regulatory processes. Oxytocin (OT) and cortisol are key neuromodulators of biological and behavioral responses, show a synergistic effect, and have been implicated in the neuropathological profile in ASD. However, they are rarely investigated together. The purpose of the pilot study was to evaluate the relationship between cortisol and OT in children with ASD under baseline and physiological stress (hydrocortisone challenge) conditions. Arginine vasopressin (AVP), structurally similar to OT, was also examined.

**Methods:**

A double-blind, placebo-controlled, randomly assigned, crossover design was employed in 25 children 8-to-12 years with ASD (*N* = 14) or typical development (TD, *N* = 11). A low dose of hydrocortisone and placebo were administered via liquid suspension. Analysis of variance (ANOVA) was used to examine the within-subject factor “Condition” (hydrocortisone/placebo) and “Time” (pre and post) and the between-subject factor “Group” (ASD vs. TD). Pearson correlations examined the relationship between hormone levels and clinical profile.

**Results:**

There was a significant Time × Condition × Group interaction *F *(1.23) = 4.18, *p* = 0.05 showing a rise in OT during the experimental condition (hydrocortisone) and a drop during the placebo condition for the TD group but not the ASD group. There were no group differences for AVP. Hormone levels were associated with social profiles.

**Conclusions:**

For the TD group, an inverse relationship was observed. OT increased during physiological challenge suggesting that OT played a stress-buffering role during cortisol administration. In contrast for the ASD group, OT remained unchanged or decreased during both the physiological challenge and the placebo condition, suggesting that OT failed to serve as a stress buffer under conditions of physiological stress.

While OT has been tied to the social ability of children with ASD, the diminished moderating effect of OT on cortisol may also play a contributory role in the heightened stress often observed in children with ASD. These results contribute to our understanding of the growing complexity of the effects of OT on social behavior as well as the functional interplay and differential regulation OT may have on stress modulation.

## Background

While many children find social interaction stress reducing, children with autism spectrum disorder (ASD) often find social interaction stress-inducing [1–3]. An appropriate response of the primary stress system, the limbic-hypothalamic-pituitary-adrenal (LHPA) axis, is essential for biological, behavioral, and psychosocial well-being. Conversely, inappropriate responsivity of the stress system may result in impairments in development and contribute to a variety of endocrine, metabolic, autoimmune, psychiatric, and social disorders [4]. However, variations in stress threshold and underlying regulatory mechanisms may contribute to differences in responsivity. In regard to ASD, social stress may be highly influenced by contextual (e.g., peers [5]) and neurohormonal (e.g., oxytocin) factors [6].

Social behavior is complex and so too is the socioemotional profile of individuals with autism. Since Kanner’s [7] earliest accounts of autism, a diverse set of social and emotional response patterns have been described. Yet, years later, the underlying mechanisms are elusive that may be contributing to the atypical social responses that are central to this disorder. The role of hormones is to assist in the regulation, drive, and adaptation of the individual to the dynamic internal and external environment, including the social world. Key hormones involved in the regulation of social and stress responses, specifically oxytocin (OT) and cortisol, are both implicated in the neuropathology of autism (e.g., [8–12]).

Cortisol is the primary glucocorticoid in humans released from the adrenal cortices following activation of the LHPA axis. The system can be activated by systemic stress (physical, context-independent, life-threatening) or processive stress (psychological, context-dependent, perceived threat) [13]. Cortisol maintains a diurnal pattern and produces a variety of effects throughout the body including influences on cardiovascular function, immunity, metabolism, and neurobiology [14], which collectively allow optimal adaptation to changing environmental demands. In addition to being involved in several vital biological processes and interactions, cortisol is central to the physiological response to physiological or perceived psychological stress [13, 15]. Importantly, the LHPA axis can be influenced by social variables that can enhance or diminish the stress response [16, 17], which is highly relevant for the study of ASD, a disorder marked by impairment in social cognition, communication, and interaction [18].

Current and ongoing research has shown that children with ASD demonstrate heightened stress to various benign and novel stimuli [9, 10, 19–21] and natural social conditions [1, 22, 23]. However, there is significant variability in response patterns of cortisol in children with ASD, which led to a hypothesized Neuroendocrine Spectrum Model in which social and arousal patterns intertwine to form unique social stress profiles [2, 3]. The hyper-responsivity of the LHPA may contribute to increased anxiety, neophobia, or even chronic stress. In fact, associations have been reported between heightened cortisol levels and self-reported trait anxiety [22, 24, 25]. Collectively, research shows that physiological arousal in ASD is on a continuum of responsivity [26] and can affect social interaction patterns [1, 3, 5].

Due to the known deleterious effects of frequent and prolonged exposure to cortisol on mental and physical well-being, continued study in persons with ASD who evidence dysregulation of the LHPA axis is warranted. Moreover, the significant variability in cortisol suggests underlying differences in physiological response patterns that may be linked to neurohormonal crosstalk. It is highly plausible that cortisol, as an important modulator of biobehavioral functioning, may provide important clues as to associations with other key regulatory neurohormones, including OT, that contribute to social stress in autism.

OT is a hypothalamic peptide crucial to the formation of social bonds [27] (for a review, see [28]. Research in animal (e.g., [12, 29–32]) and human models [33–35] provides compelling evidence for the involvement of OT in mediating complex social behavior. OT plays an important role in stress buffering by reducing the responsiveness of the LHPA axis [33, 34, 36, 37]. Specifically, it appears that OT is an important moderator of stress by, in part, reducing activation of the medial amygdala [38] and modulating activation of the amygdala in response to facial expressions [39], which has been found to be dysregulated in autism [40–44].

In humans, OT facilitates social approach and provides anxiolytic effects [45], especially when combined with social support as shown in studies comparing intranasal OT vs. placebo [46, 47]. This blunting effect appears short-term and specific to cortisol response [48]. Ostensibly, OT can reduce the responsivity of the LHPA axis to social stress by reducing uncertainty [39] and enhancing trust [40, 49, 50], resulting in more social approach [51]). Further, OT may restrict the LHPA by making arginine vasopressin (AVP) less reactive [52] and buffer the vasopressin-ACTH-cortisol response [53]. For some stressors, OT has been shown to reduce corticotropic releasing hormone gene expression in the paraventricular nucleus, leading to reduction in ACTH and cortisol [54]. The results support the hypothesis that OT is a key moderator of social behavior and regulator of stress reactivity [27, 55]. Although the results to date supporting a reciprocal relationship between OT and stress are compelling, some findings suggest that the interplay is complex and influenced by individual differences [47, 56] and social distress [57]. One of the roles of OT may be to motivate people to find positive social affiliations [57]. Thus, OT is influenced by individual factors associated with social affiliation and distress [58].

The neuropeptide OT has been associated with the neurobiology of autism [8, 12, 59–64]. Individuals with autism reportedly have impaired OT processing, resulting in higher levels of plasma OT-X, a precursor to the normal adult form of OT, and lower levels of OT [65–68]. In one study, elevated OT was associated with more sociality in typically developing children but was associated with less social behavior in children with autism, especially those characterized as aloof. [67] Several studies have shown genetic associations between the oxytocin receptor gene (OXTR) and autism [60, 69–75].

Recent reports suggest that OT may be a novel therapeutic target for treating social impairments of autism [58, 76–80]. OT treatments have been shown to increase the ability of adults with autism to evaluate emotional significance in speech [81, 82], reduce repetitive behaviors [11], and improve emotion recognition in youth [44, 83, 84] and adults [85] with ASD.

There are many levels at which OT might impact on autism, including through the capacity to influence both sociality and to downregulate stress reactivity of the LHPA axis [27, 86]. Given that OT interacts to suppress cortisol and responses to psychosocial stress [46], and is currently being considered as a possible target treatment [58, 77, 79, 80], with potential long-term risk [87], an integrated study examining the relationship between these key regulatory hormones, cortisol, and OT was conducted.

Based in part on the previous studies reviewed, it is likely that these regulatory neurohormones, individually and collectively, contribute to the variability and severity of social stress profiles in children with ASD. Thus, the current study was designed to investigate cortisol and OT under baseline, pharmaceutical hydrocortisone challenge (HCORT), and placebo (PLACEBO) conditions in children 8 to 12 years of age with high-functioning ASD or typical development (TD). It was hypothesized that children in both groups would show comparable baseline values of cortisol and an increase in cortisol following hydrocortisone challenge; however, it was predicted that children with ASD as a group would show greater variability in cortisol expression. In regard to OT, it was predicted that children in both groups would show comparable baseline values; however, children with TD would show an increase in OT to HCORT and a lack of change or decrease in OT to the placebo (PLACEBO). Conversely, it was predicted that children with ASD would show a lack of change or decrease in OT following both HCORT and PLACEBO suggesting that endogenous OT would fail to serve as a stress buffer. Finally, it was predicted that cortisol and OT values would be negatively and positively correlated with the social responsiveness profiles, respectively.

## Methods

### Ethics, consent, and permission

The Vanderbilt Institutional Review Board approved the study. Informed written consent was obtained from both parents, and assent was obtained from child participants prior to inclusion in the study.

### Participants

Inclusion criteria required all participants to be free of prescribed medications and no known allergic reaction to or current use of hydrocortisone or related pharmaceutical agents. Participation in the study required three visits to the University. During visit 1, the diagnostic and psychological measures described below were administered and the results are presented in Table 1. All enrolled participants were provided with a research letter containing the results from the standardized measures.

**Table 1:** Demographic and diagnostic information for children with typical development (TD) and autism spectrum disorder (ASD)

	TD		ASD				
Measure	Mean	SD	Mean	SD	*t*	*df*	*p*
AGE	9.37	1.58	9.70	1.93	−0.47	24	0.64
ADOS			13.82	4.56			
SCQ	2.27	2.00	19.91	6.63	−8.45	20	0.00
SRS	45.82	6.60	77.73	12.08	−7.69	20	0.00
WASI	121.8	15.48	117.0	39.27	0.38	21	0.71

*ADOS* Autism Diagnostic Observation Schedule, *ASD* autism spectrum disorder, *SCQ* Social Communication Questionnaire, *SD* standard deviation, *SRS* Social Responsiveness Scale, *TD* typical development, *WASI* Wechsler Abbreviated Scale of Intelligence

Inclusion in the typically developing group required an absence of any known neurological, medical, or psychiatric condition, an IQ ≥70 [88], and a score <10 on the Social Communication Questionnaire [89] described below. Inclusion in the ASD group required a confirmed ASD diagnosis based on the Diagnostic and Statistical Manual-5 [18] established by (1) a previous diagnosis by a psychologist, psychiatrist, or behavioral pediatrician with autism expertise; (2) current clinical judgment (BAC); and (3) corroborated by the Autism Diagnostic Observation Schedule (ADOS) [90], administered by research-reliable personnel. Participants with ASD also had to have an IQ >70 [88].

Independent sample *t* tests were conducted to assess mean, standard deviations, and potential between-group differences on the demographic variables. There were no significant differences between the groups based on age or IQ (see Table 1). As expected, there were significant differences between groups on both social functioning questionnaires (i.e., SCQ and SRS).

### Diagnostic and psychological measures

*Autism Diagnostic Observation Schedule* (ADOS [90]) is a semi-structured interview designed to assess behaviors indicative of autism. A score of ≥8 on the social communication domain is required.

*Wechsler Abbreviated Scale of Intelligence (WASI* [88] is a measure of cognitive ability that will be used to obtain a quick, reasonable estimate of a child’s intellectual functioning (IQ ≥70 required).

*Social Communication Questionnaire* (SCQ) [89] is a screening tool for ASD. Scores of 15 or higher are highly suggestive of ASD.

The *Social Responsiveness Scale* (SRS) [91] is a parent-report measure covering several areas of behavior characteristics of autism with good temporal stability (males *r* = .85, females *r* = .77) and internal consistency (Cronbach’s *α* > .90).

### Hydrocortisone vs. placebo challenge protocol

#### Rationale

To specifically evaluate the relationship between cortisol and OT, participants were exposed to a single-dose hydrocortisone (pharmaceutical cortisol) challenge. Although dexamethasone has been explored in children with autism [92–95], this approach is not warranted in the current investigation. Dexamethasone is a synthetic glucocorticoid 20 to 30 times more potent than hydrocortisone that is frequently used to evaluate the function of the LHPA axis [96, 97]. Furthermore, the actions of dexamethasone in the central nervous system are of longer duration (plasma half-life), which can result in behavioral, psychological, and cognitive changes including adverse effects [98]. For this investigation, the aim was to evaluate the relationship between hormones rather than assess the functionality of the negative feedback mechanisms of the LHPA axis. Finally, while glucocorticoids, such as hydrocortisone, can impact social behavior such as reduction in phobic social fear [99], the dose in the current study was comparatively low.

#### Hydrocortisone study protocol

A double-blind, placebo-controlled, randomly assigned, crossover design was employed. The protocol was conducted at the Pediatric Clinic Research Center at Vanderbilt University over two visits with 1-week intervals. The order of HCORT or PLACEBO was randomly determined by Vanderbilt Investigational Drug Service (IDS) pharmacy. The investigative team and study participants were blind to order assignment. Following a health status check by medical staff, participants were administered hydrocortisone during one visit (1) a low dose of hydrocortisone and (2) placebo administration prepared by IDS. Hydrocortisone (5 mg per m2) or placebo was administered via liquid suspension one syringe of liquid formulation (15 mL). Blood samples were drawn by a pediatric phlebotomist.

#### Dose

The dose of drug and placebo was determined by the participant’s BSA. Prior to administration, height and weight were assessed to calculate body surface area (m^2^) to determine appropriate hydrocortisone dose (mg/day) using the Mosteller method [100] BSA (m^2^) = ([Height(cm) × Weight(kg)}/3600)^1/2^. Children determined to be obese (have a bsa > the 95th %) were excluded from participation in the study [101]. The administration of a single dose of hydrocortisone was administered in one dose level, which is considered to be mild (5 mg per m^2^) and placebo. This is considered a low level of hydrocortisone, and behavioral changes were expected to be subtle. Average pediatric doses range from 10 to 25 mg/day [102]. In pediatric patients, the initial dose of hydrocortisone for various diseases is considerably higher than what was administered in the current study with levels ranging from 20 to 240 mg per m^2^ bsa per day. Demonstrable effects are detectable within 1 h; thus, sample collection of cortisol and OT were taken at baseline and at 60-min post administration.

#### Sample collection

For each blood draw, 6 ml was collected. Repeat blood collection occurred at (1) baseline, approximately 15 min after arrival and acclimation to the clinic setting and immediately before hydrocortisone/placebo administration and (2) 60 min after the administration of the pharmaceutical agent. Blood was collected on ice and centrifuged at 4 °C, 3300 rpm for 12 min. Plasma was then stored in a −80 °C freezer until assay. Serum and salivary samples from 14 children with ASD (12 males, 2 females) and 11 neurotypical (10 males, 1females) children were completed for hydrocortisone and placebo conditions.

#### Oxytocin assay

The oxytocin assay (Enzo Life Sciences, Farmingdale, NY) has been validated for various species including humans [103–105]. The assay was conducted at a dilution of 1:5 similar to other human studies. Samples were collected at approximately the same time of day (1:00 to 4:00 p.m.). The measurement of peripheral OT has been controversial, in that its levels in plasma are not always correlated with levels in cerebrospinal fluid [106, 107]. However, in many recent studies, peripheral measures have been used as a valuable reflection of OT system activity [108–110]. In addition, there is disagreement as to whether or not plasma samples should be extracted before using commercial enzyme immunoassay kits. The proponents of extraction believe that the high levels measured in unextracted samples indicate that these readings include other substances in addition to OT [111, 112]. Proponents of non-extracted samples believe that extraction removes a portion of the OT which is sequestered in plasma proteins [113]. Recent unpublished findings in mass spectrometry support this view showing high levels of OT closer to those in unextracted samples [114]. The c.v.s for assays were intra-assay, 2.45 %, and inter-assay, 8.61 %.

#### Cortisol sample collection and assays

Salivary cortisol collection has been previously described for home salivary samples (4× per day for 3 days for 2 weeks) and at arrival, baseline, 20, 40, and 60 min post stress exposure [9, 10]. Assays were performed using coated tube radioimmunoassay RIA kits (Siemens Medical Solutions Diagnostics, Los Angeles) and modified to accommodate overall lower levels of cortisol in human saliva relative to plasma.

#### AVP assay

The AVP assay (Enzo Life Sciences, Farmingdale, NY) was conducted at a dilution of 1.1 similar to other human studies in the lab [61] and comparable to the field which usually use dilutions between 1:1 and 1:2 [115, 116]. As noted above, samples were collected at approximately the same time of day (1:00 to 4:00 p.m.). The intra-assay cv for the AVP assay is 4.63 %. The inter-assay cv is 11.60 %.

### Statistical analysis

Independent sample *t* tests were conducted to assess mean, standard deviations, and potential between-group differences on the dependent variables. Analysis of variance (ANOVA) was the primary statistical approach to determine within and between subject effects on the dependent variables (OT, cortisol, and AVP). The ANOVA model included the within subject factors “Condition” (hydrocortisone/placebo) and “Time” (pre and post) and the between-subject factor “Group” (ASD vs. TD). Due to skewed distribution, salivary and plasma cortisol were log transformed prior to inclusion in the model. Paired sample *t* tests were conducted to specify the meaning of statistically significant interactions involving Condition and Group. Finally, to examine the relationships between hormone concentrations and subject characteristics, Pearson product moment correlations were conducted.

## Results

Using independent sample *t* tests, there were no precondition baseline between-group differences for OT, plasma cortisol, and salivary cortisol (all *p* > 0.05; see means and standard deviations in Table 2). However, there was a trend for baseline differences for AVP *t*(23) = −2.01, *p* = 0.06 showing slight elevation in the ASD compared to the TD group (see Table 2).

**Table 2:** Means and standard deviations for oxytocin (OT) (pg/ml), cortisol (ng/ml), and arginine vasopressin (AVP) (pg/ml)

Measure				
Oxytocin	TD mean	TD SD	ASD mean	ASD SD
OT pre-HCORT	2009.92	813.83	2340.36	1145.38
OT post-HCORT	2148.55	777.79	2140.67	951.47
OT pre-PLACEBO	2566.98	921.75	2199.97	822.24
OT post-PLACEBO	2089.01	670.12	2074.19	886.10
Cortisol-P plasma				
Cortisol-P pre-HCORT	0.91	0.30	0.93	0.16
Cortisol-P post-HCORT	1.53	0.44	1.68	0.19
Cortisol-P pre-PLACEBO	0.85	0.13	0.95	0.11
Cortisol-P post-PLACEBO	0.75	0.19	0.91	0.14
Cortisol-S saliva				
Cortisol-S pre-HCORT	0.91	0.30	0.93	0.16
Cortisol-S post-HCORT	1.54	0.44	1.68	0.19
Cortisol-S pre-PLACEBO	0.85	0.13	0.95	0.11
Cortisol-S post-PLACEBO	0.75	0.19	0.91	0.14
Arginine vasopressin AVP				
AVP pre-HCORT	1.94	0.26	2.16	0.28
AVP post-HCORT	2.05	0.27	2.12	0.19
AVP pre-PLACEBO	1.98	0.24	2.23	0.28
AVP post-PLACEBO	2.11	0.37	2.14	0.32

**Fig. 1:**
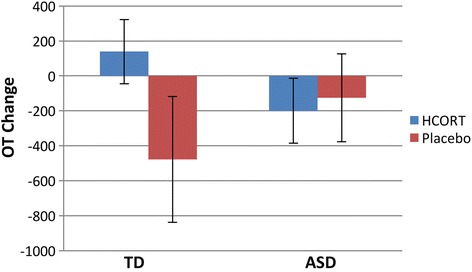
Mean oxytocin change across conditions (hydrocortisone and placebo) and group (ASD and TD). The mean OT change between post minus prehydrocortisone (HCORT, *blue*) and placebo (*red*) administration between children with ASD and TD. In the TD group, there was a significant change in OT during HCORT compared to placebo condition. In contrast, the ASD group did not show a significant change in OT between the conditions

Using ANOVA to examine Time, Condition, and Group effects, for OT, there was a significant difference for Time *F*(1,23) 8.95, *p* = 0.007) indicating the expected difference between pre and post for both groups based on the HCORT levels. As predicted, there was a significant Time × Condition × Group interaction *F*(1.23) = 4.18, *p* = 0.05 in OT (see Fig. 1).

To examine Condition effects, we conducted a paired *t* test on the change scores of OT between conditions by each group. There was a Condition effect in the TD group (mean condition differences = 616.6; SD = 929.89, *t*(10) = 2.0; *p* = .05), but not in the ASD group (mean condition differences = −73.91, SD = 760.56, *t*(13) = −.36, *p* = .72). Given that the condition difference was in opposite directions between diagnostic groups, it is clear that the condition effect is significantly different between diagnostic groups.

Using ANOVA, the AVP results revealed no Time effect *F*(1,23) 0.23, *p* = 0.64. Moreover, there was no Time × Group *F*(1,23) 2.52, *p* = 0.13, Condition × Group *F*(1,23) 0.80, *p* = 0.78 or Time × Condition × Group interaction *F*(1,23) 0.26, *p* = 0.62.

**Fig. 2:**
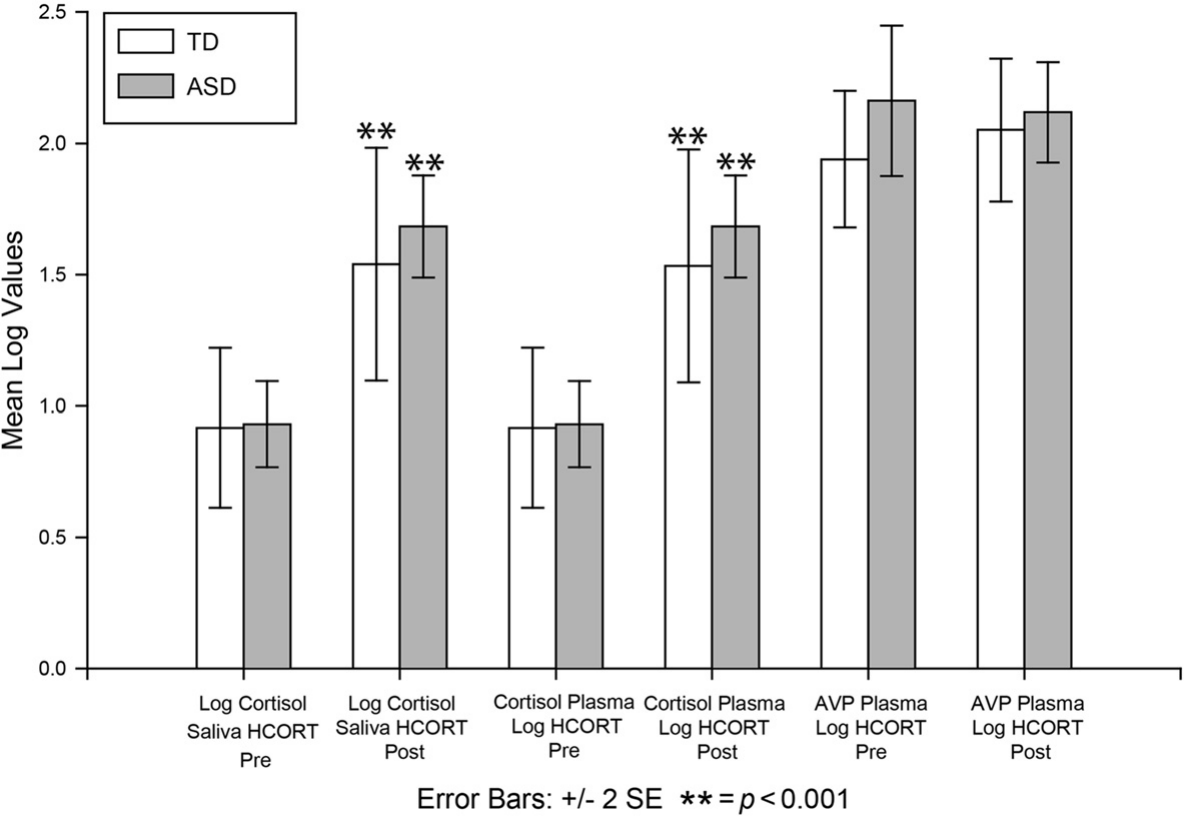
Levels pre- and posthydrocortisone (HCORT) challenge across groups. The mean log salivary and plasma cortisol and plasma arginine vasopressin (AVP) are shown for pre- and post-HCORT administration in children with typical development (TD; *white*) and autism spectrum disorder (ASD; *gray*)

Additional statistical approaches were employed to further explore the measurement and change of cortisol. Since hydrocortisone is pharmaceutical cortisol, analyses focused on confirming expected differences across the conditions and demonstrating the strong relationships between plasma and salivary cortisol. Paired sample *t* tests of logged data showed a significant difference in plasma cortisol *t*(24) −8.21, *p* = 0.001, and salivary cortisol *t*(24) −8.23, *p* = 0.001 following HCORT indicating an expected rise in circulating cortisol in response to administration of hydrocortisone. However, there was not a significant difference in cortisol following PLACEBO *t*(24), 1.82, *p* = 0.08. Thus, plasma and salivary cortisol rose in response to the pharmaceutical challenge but remained unchanged showing comparable values across the groups for the PLACEBO condition (see Fig. 2).

Levene’s Test for Equality of Variances was used to examine variability for each of the hormones. Results revealed a significant difference for cortisol plasma HCORT *F *(1,23) = 4.31, *p* = 0.05; however, there were no significant between-group differences on the salivary cortisol, OT, or AVP levels.

**Fig. 3:**
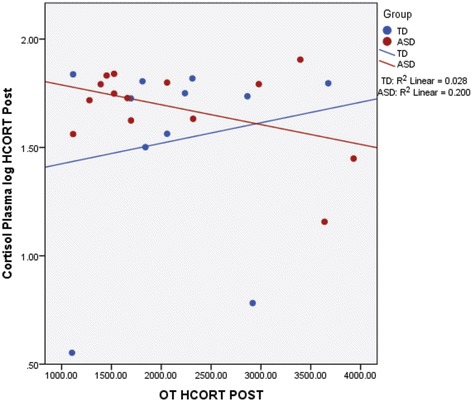
Correlations between plasma cortisol and oxytocin (OT) following administration of hydrocortisone (HCORT) in children with TD (*blue*) and ASD (*red*). As shown, there are opposing relationships between the groups. Within-group correlations in the ASD children revealed a negative correlation between OT and cortisol following HCORT administration

Pearson product moment correlations were conducted to examine associations between the hormones. For the total sample, plasma and salivary cortisol were highly correlated *r* = .99, *p* = 0.001, as expected. There were no significant correlations between OT, cortisol, and AVP during baseline, pharmaceutical challenge, or placebo (all *p* > 0.05). In regard to within-group correlations, for the ASD group, the only significant comparison was OT and cortisol following HCORT *r* = −.47, *p* = 0.05. As Fig. 1 illustrates, the two groups showed opposing directional effects. For the TD group, the only significant correlation was between OT and cortisol at baseline *r* = .53, *p* = 0.05 (see Fig. 3).

In regard to hormone levels and symptom profile, there were a few correlations. In the TD group, the SRS (impairment scale of social functioning) was positively associated with baseline plasma cortisol (*r* = .68, *p* = 0.02) and AVP (*p* = 0.03). In the ASD group, there was a modest negative trend for the SRS and AVP baseline (*r* = −.54, 0.08). There were no significant correlations between the SRS and post hydrocortisone or placebo administration (all *p* > 0.05).

## Discussion

The current study was designed to investigate cortisol, OT, and AVP under baseline, pharmaceutical HCORT, and PLACEBO conditions in children with ASD and TD. In regard to OT, it was predicted that children in both groups would show comparable baseline values; however, children with TD would show an increase in OT to HCORT and a decrease in OT to the PLACEBO. Conversely, it was predicted that children with ASD would show a decrease in OT following both HCORT and PLACEBO suggesting that endogenous OT would fail to serve as a stress buffer. Finally, it was predicted that cortisol and OT values would be negatively and positively correlated with the social responsiveness profiles, respectively.

There were no between-group baseline differences for OT which do not support the oxytocin-deficit hypothesis model in autism [40, 67, 117] as children with and without ASD showed comparable baseline OT plasma concentration prior to HCORT and PLACEBO supporting our hypothesis. There were also no significant differences in AVP, which is similar to Miller and colleagues [61] that challenged the prevalent notion that these neuropeptides are lower in children and adolescents with ASD. The findings are also in agreement with Parker and colleagues [118] that showed no baseline plasma differences in OT in a large sample of participants indicating similar circulating concentration between children with ASD and non-ASD children.

Following the administration of hydrocortisone, an inverse relationship was observed for the typically developing children such that OT increased during physiological challenge and remained stable or decreased during placebo administration. As predicted, these findings suggest that OT played a stress-buffering role during cortisol administration. The decrease in OT during the placebo condition supports the idea that the lab procedures were not inherently stress producing. Yet for the ASD group, OT remained stable or decreased during both the physiological challenge and the placebo condition, suggesting that OT failed to serve as a stress buffer under conditions of physiological stress.

While OT has been tied to the social ability of children with ASD [119], the diminished moderating effect of OT may play a contributory role in the heightened stress often observed in children with ASD especially during social interactions [1–3]. In other words, in addition to experiencing heightened stress in response to novel and changing situations, it appears that oxytocin does not assist in ameliorating stress once activated. Thus, the results contribute to the growing complexity in our understanding of the effects of OT on social behavior including individuals on the autism spectrum [87].

Even though AVP is structurally similar to OT, there were no significant differences between the groups, over time, or across conditions. Additionally, there were no significant correlations between AVP and the other hormones. It is important to note that there was significant variability in the samples, especially in the TD group during the placebo condition. It is possible that this larger variability and rather small sample size may have prevented the detection of plausible differences.

It was hypothesized that children in both groups would show comparable baseline values of cortisol and an expected increase following HCORT, which was supported. Participants evidenced a significant rise in both plasma and salivary cortisol following the hydrocortisone challenge, which was not demonstrated in the placebo condition. Therefore, children with and without ASD showed an adaptive rise in peripheral concentrations of cortisol following controlled pharmaceutical administration suggesting no systemic dysregulation of the LHPA axis under such conditions.

Previous studies have shown a tendency for greater variability in the expression of cortisol in children with ASD [1, 3]. While the current findings showed significant variability in plasma cortisol following the administration of hydrocortisone, it was observed in the TD not the ASD group. It may be the case that context and the type of stressor play an important role in the individual expression of cortisol between and within groups as has been previously suggested [1, 3].

In addition to the primary analyses described above, associations between the hormones were explored. While there was an inverse relationship between OT and cortisol following the administration of hydrocortisone in the ASD group, it did not reach significance. Nevertheless, it appears to be consistent with the observed interaction between the groups, time, and condition such that the children with ASD seemed to show a down regulation of OT when physiologically challenged which was opposite of the TD group. There was also a trend level positive correlation at baseline between cortisol and OT suggesting the expected synergistic relationship between arousal and stress buffering between these hormones.

The current study induced a stress response via pharmaceutical challenge therefore removing individual differences based on perceived stress. This allowed for the examination of regulatory hormonal response under carefully controlled dose and response conditions without the impact of influential processive factors [13]. Moreover, the randomized, double-blind, crossover design limited bias and enabled direct comparison between the participants across comparable physiological conditions. As a result, alternative explanations not related to the experimental condition (HCORT) such as history, testing, or statistical effects (e.g., regression to the mean) [120] were minimized or removed. To our knowledge, this is the first experiment in which cortisol levels were experimentally manipulated in order to methodically examine the regulation of oxytocin during stress induction.

Despite the strengths of the study, there are limitations to acknowledge. Most notably, the pilot study included a rather small sample of high-functioning participants with ASD and TD within a rather narrow age range. Thus, the generalizability of the findings may be limited based on functioning level and age. We also acknowledge that the measurement of peripheral OT, AVP, and cortisol is only a proxy for underlying regulatory processes. The extent to which these peripheral hormones individually and collectively represent the underlying physiological complexity is unclear. In particular, there remains a controversy in the field regarding OT with some proponents insisting on extraction methods [111, 112]. In consideration of this debate, our decision to not extract OT may be considered an additional limitation of the study.

## Conclusions

These data build on previous models showing that the interplay between OT and stress is complex and influenced by many factors {Heinrichs, 2008 #797; Taylor, 2006 #736; Turner, 1999 #794}. These findings provide the foundation and justification for future work exploring biobehavioral influences to elucidate the heterogeneity in social functioning and stress modulation in autism. Moreover, the findings emphasize the complexity of not only the social presentation of children with ASD but also the underlying associated hormonal profile that warrants critical consideration. The future study of neurohormone regulation in general, including the modulatory role of OT on physiology, is essential especially as frequent and unregulated treatment with OT is emerging [87]. Moreover, the impact of other factors, such as context and social support will be important to explore as plausible mediating variables in the complex interplay between oxytocin and cortisol regulation.

## Abbreviations

ADOS: Autism Diagnostic Observation Schedule
ASD: autism spectrum disorder
AVP: arginine vasopressin
HCORT: hydrocortisone challenge
IDS: Investigational Drug Service
LHPA: limbic hypothalamic pituitary adrenal axis
OT: oxytocin
SCQ: Social Communication Questionnaire
SRS: Social Responsiveness Scale
WASI: Wechsler Abbreviated Scale of Intelligence
TD: typical development
